# The Antidiabetic Drug Lobeglitazone Protects Mice From Lipogenesis-Induced Liver Injury via Mechanistic Target of Rapamycin Complex 1 Inhibition

**DOI:** 10.3389/fendo.2018.00539

**Published:** 2018-09-21

**Authors:** Yu Seol Lee, Jeong Su Park, Da Hyun Lee, Dong-Kyu Lee, Sung Won Kwon, Byung-Wan Lee, Soo Han Bae

**Affiliations:** ^1^Brain Korea 21 PLUS Project for Medical Science, Yonsei University, Seoul, South Korea; ^2^Severance Biomedical Science Institute, Yonsei Biomedical Research Institute, Yonsei University College of Medicine, Seoul, South Korea; ^3^Research Institute of Pharmaceutical Sciences, Seoul National University, Seoul, South Korea; ^4^College of Pharmacy, Seoul National University, Seoul, South Korea; ^5^Graduate School, Yonsei University College of Medicine, Seoul, South Korea; ^6^Institute of Endocrine Research, Yonsei University College of Medicine, Seoul, South Korea; ^7^Division of Endocrinology and Metabolism, Department of Internal Medicine, Yonsei University College of Medicine, Seoul, South Korea

**Keywords:** lobeglitazone, NAFLD, thiazolidinedione, mTORC1, ER stress

## Abstract

Non-alcoholic fatty liver disease (NAFLD) is a metabolic disorder closely linked with type II diabetes (T2D). The progression of NAFLD is associated with the induction of lipogenesis through hyperactivation of the mechanistic target of rapamycin complex 1 (mTORC1) pathway. An increase in lipogenesis induces endoplasmic reticulum (ER) stress and accelerates oxidative liver injury in the pathogenesis of NAFLD. Lobeglitazone, one of thiazolidinediones (TZDs), is used as an antidiabetic drug to lower serum glucose level through an increase in insulin sensitivity. It is known to improve pathological symptoms in animals and humans with NAFLD. However, the underlying molecular mechanism of the protective effects of lobeglitazone against NAFLD has not been elucidated. Here, we show that under the physiological condition of acute lipogenesis, lobeglitazone inhibits hepatic lipid synthesis, the subsequent ER stress, and ω-oxidation of fatty acids by inhibiting the mTORC1 pathway. As a result, lobeglitazone protected mice from lipogenesis-induced oxidative liver injury. Taken together, lobeglitazone might be a suitable drug for the treatment of patients with diabetes and NAFLD.

## Introduction

Non-alcoholic fatty liver disease (NAFLD), encompassing a spectrum from simple steatosis to non-alcoholic steatohepatitis (NASH), is associated with metabolic disorders such as obesity, type II diabetes (T2D), and insulin resistance (1–3). Insulin resistance induces hepatic lipogenesis, precipitating lipid accumulation and oxidative stress in the liver (4).

Hepatic lipogenesis is increased by expression of the basic helix-loop-helix-leucine zipper (bHLH-Zip) transcription factors, such as sterol regulatory element-binding protein-1c (SREBP-1c) and carbohydrate response element binding protein (ChREBP) (1, 5, 6). SREBP-1c is positively regulated by hyperactivaiton of mechanistic target of rapamycin complex 1 (mTORC1) manifested in NAFLD, which promotes lipid synthesis (7–9). Consequently, excess lipids induce stress in the endoplasmic reticulum (ER) stress and the ω-oxidation of fatty acids; in turn, this leads to oxidative stress in the liver. Thus, recent studies have revealed that targeting of hepatic lipogenesis is a prospective therapeutic strategy for the treatment of NAFLD (10).

Recently, several studies reported that thiazolidinediones (TZDs), antidiabetic drugs for the treatment of T2D, prevent the development of NAFLD in clinical data and *in vivo* experiments (11–13). Lobeglitazone is a recently developed TZD that increases insulin sensitivity through the promotion of binding between insulin and its receptor in adipose tissue (14, 15). Similar to other TZDs, lobeglitazone ameliorated insulin resistance and hepatic steatosis (16, 17); however, the detailed mechanism of its anti-steatotic effects remains unclear. Thus, we investigated the effects of lobeglitazone and molecular mechanisms of its action in the progression of NAFLD.

## Materials and methods

### Antibodies and reagents

Antibodies to S6, phosphorylated S6, phosphorylated P70S6K, 4EBP1, phosphorylated 4EBP1, Akt, phosphorylated Akt, mTOR, and phosphorylated mTOR were purchased from Cell Signaling Technologies. The antibody to P70S6K was purchased from Cusabio. Lobeglitazone was generously supplied by Chong Kun Dang Pharmaceutical Corporation (Seoul, Republic of Korea) and dimethyl sulfoxide (DMSO) was purchased from Sigma Aldrich.

### Animals

Male C57BL/6J mice, 9–11 weeks of age, were purchased from Japan SLC, Inc. (Hamamatsu, Japan). These animals were randomly grouped into one of three types: animals administered vehicle and fed a normal chow diet without fasting; animals administered vehicle and fed a high-carbohydrate diet, fat-free diet (HCD) after a 24 h fast; and animals administered lobeglitazone or rapamycin and fed a HCD after a 24 h fast. All mice were given free access to water and food in an environment maintained at 23 ± 2°C, 12/12 h light dark cycle, and between 50 and 70% humidity. Before vehicle, lobeglitazone (1 mg/kg), or rapamycin (8 mg/kg) was administered by oral gavage, the mice were fasted for 0 or 24 h and then given access to food. After 12 h of feeding, the mice were sacrificed. All animal experiments were approved by the Animal Care and Use Committee of the Yonsei University College of Medicine.

### Measurement of serum ALT levels

The blood obtained from heart of mice was incubated at room temperature for 1 h, and then centrifuged at 3,000 rpm for 10 min. The resulting supernatants were collected in new tube and measured by using a colorimetric assay kit (BioAssay Systems) to qunatifiy serum alanine aminotransferase (ALT) level.

### Immunoblot analysis

For immunoblot analysis, mice livers or primary hepatocytes were homogenized in analysis buffer [50 mM Tris-HCl (pH 7.5), 150 mM NaCl, 1 mM 4-(2-aminoethyl)-benzensulfonyl floride, 1% NP-40, and protease inhibitors]. The cell lysates were centrifuged and the resulting supernatants were collected and measured by using the Bradford assay (Bio-Rad). The cell lysates were separated by SDS-polyacrylamide gel electrophoresis and transferred onto a polyvinylidene difluoride membrane. The membranes were blocked, incubated with primary antibodies overnight, and then incubated with horseradish peroxidase (HRP)-conjugated secondary antibodies. Protein staining was visualized by the application of enhanced chemiluminescence reagents (Thermo Scientific), and the abundance of phosphorylated forms of the protein was quantitated by densitometric analysis of the immunoblots and normalized to the total form of the protein, respectively.

### Quantitative RT-PCR analysis

Total RNA was isolated from liver tissue by using Trizol reagent. The RNA was reverse transcribed into cDNA by using a cDNA synthesis kit (TAKARA). The resulting cDNA was subjected to real-time PCR analysis using SYBR® Green and an ABI PRISM 7700 system (Applied Biosystems). Ribosomal RNA (18S) was used as an internal control. The sequences of primers for mouse cDNAs (forward and reverse, respectively) were as follows: FAS, 5′-GCTGCGGAAACTTCAGGAAAT-3′ and 5′-AGAGACGTGTCACTCCTGGACTT-3′; SCD-1, 5′-CCGGAGACCCCTTAGATCGA-3′ and 5′-TAGCCTGTAAAAGATTTCTGCAAACC-3′; ACC1, 5′-TGGACAGACTGATCGCAGAGAAAG-3′ and 5′-TAGCCTGTAAAAGATTTCTGCAAACC-3′; LCE, 5′-TGTACGCTGCCTTTATCTTTGG-3′ and 5′-GCGGCTTCCGAAGTTCAA-3′; GPAT, 5′-CAACACCATCCCCGACATC-3′ and 5′-GTGACCTTCGATTATGCGATCA-3′; ATF4, 5′-ACACAGCCCTTCCACCTC-3′ and 5′-CACGGGAACCACCTGGAG-3′; EDEM, 5′-GGATCCCCTATCCCTCGGGT-3′ and 5′-GTTGCTCCGCAAGTTCCAG-3′; TRB3, 5′-CTCTGAGGCTCCAGGACAAG-3′ and 5′-GGCTCAGGCTCATCTCTCAC-3′; Grp78, 5′-GAAAGGATGGTTAATGATGCTGAG-3′ and 5′-GTCTTCAATGTCCGCATCCTG-3′; Cyp4α10, 5′-ACACTGCTCCGCTTCGAACT-3′ and 5′-CAGCACAAGTCGGGCTAAGG-3′; Cyp4α14, 5′-CCCCTCTAGATTTGCACCAGAT-3′ and 5′-TCCCAATGCAGTTCCTTGATC-3′; CPT-1, 5′- CACCAACGGGCTCATCTTCTA-3′ and 5′-CAAAATGACCTAGCCTTCTATCGAA-3′; CHOP, 5′-CATACACCACCACACCTGAAAG-3′ and 5′- CCGTTTCCTAGTTCTTCCTTGC-3′; SREBP-1c, 5′-GGAGCCATGGATTGCACATT-3′ and 5′-GGCCCGGGAAGTCACTGT-3′; ChREBP, 5′-GTCCGATATCTCCGACACACTCTT-3′ and 5′- CATTGCCAACATAAGCGTCTTCTG-3′; GSTA1, 5′-TGCCCAATCATTTCAGTCAG-3′ and 5′-CCAGAGCCATTCTCAACTA-3′; NQO1, 5′-TTCTCTGGCCGATTCAGAG-3′ and 5′-GGCTGCTTGGAGCAAAATAG-3′; HO-1, 5′-GAGCAGAACCAGCCTGAACTA-3′ and 5′-GGTACAAGGAAGCCATCACCA-3′; PPARα, 5′-TCGGCGAACTATTCGGCTG-3′ and 5′-GCACTTGTGAAAACGGCAGT-3′; PPARγ, 5′-CTCTCAGCTGTTCGCCAA-3′ and 5′-CACGTGCTCTGTGACGATCT-3′; 18S, 5′-CGCTCCCAAGATCCAACTAC-3′ and 5′-CTGAGAAACGGCTACCACATC-3′.

### Histological analysis

The liver tissues of mice were fixed in 10% neutral-buffered formalin solution, embedded in paraffin, and sliced into 5-μm sections. The liver sections were subjected to hematoxylin and eosin (H&E) staining.

### TUNEL analysis

For the analysis of apoptosis in the liver tissue in mice, the Click-iT Plus TUNEL assay kit (Life Technologies, C10617) was used in accordance with the manufacturer's instructions. The fluorescence signals were detected by using a confocal microscope (LSM 700, Carl Zeiss). The frequency of apoptotic cells in the liver sections was quantified by the determination of the percentage of TUNEL-positive cell in five random microscopic fields per specimen.

### Fatty acid profiling

To each 10 mg aliquot of liver tissue, 1 mL of 7% methanolic HCl (Sigma-Aldrich) and 5 μg of non-adecanoic acid (C19:0, Sigma-Aldrich) were added as an internal standard (18). Tissue samples were placed on dry ice and then homogenized by ultrasonication (30% amplitude; 4 s pulse/1 s pause) for 30 min by using Vibra-cell ultrasonic liquid processor (VCX130, Sonics & Materials, Inc.) (19). Lipid hydrolysis and acidic transmethylation of fatty acids in homogenates were performed at 100°C for 2 h. After the samples were cooled to room temperature, the methylated fatty acids were extracted three times with 1 mL of hexane. The collected organic layer was evaporated under nitrogen and reconstituted into 100 μL of hexane. The fatty acids were then quantified by using GC-MS (GCMS-QP2010, Shimadzu) equipped with a DB-5MS capillary column (30 m × 0.25 mm, 0.25 μm, Agilent). The injection volume was 1 μL in 1:2 split mode and helium was used as a carrier gas at a constant flow rate of 1 mL/min. The injection temperature was 270°C and the column temperature program for separation was as follows: an initial temperature of 70°C for 1 min, increased to 150°C at 20°C/min, increased to 180°C at 6°C/min, increase to 220°C at 20°C/min, held for 1 min, increased to 240°C at 4°C/min, and held for 17 min, over a total period of 35 min. Electron impact (EI) at 70 eV and a source temperature of 200°C was used for compound ionization. The mass detection range between 40 and 500 m/z, with a scan rate of 2,500 s^−1^. For identification, the retention index (RI) was measured by using an alkane mixture (C7-C40, Sigma-Aldrich) and each peak in the spectrum was compared with the NIST mass spectral library (NIST08). The peak area of fatty acids was integrated by using the total ion chromatogram and normalized to the internal standard.

### Diglyceride (DG) and triglyceride (TG) profiling

The internal standards, 1 μg DG (12:0/12:0) and 1 μg TG (17:0/17:0/17:0), were spiked in 10 mg of tissue sample (20). Each aliquot was immersed in chloroform/methanol/water solution (2:5:2, v/v/v, J.T. Baker) and extracted ultrasonically by using the same parameters as for fatty acid profiling. Thereafter, liquid-liquid extraction (LLE) was performed by the addition of 1 mL of chloroform and water. The organic layer was transferred to a glass vial and the LLE was repeated three times. The lipid extracts were filtered through a 0.2 μm PTFE syringe filter (Advantec) and the filtrates were evaporated to dryness under nitrogen gas. Finally, the lipids were reconstituted in 200 μL of isopropanol. The prepared samples were injected into Agilent 1290 HPLC equipped with BEH C18 column (2.1 × 100 mm, 1.7 μm, Waters). The lipids were separated by using a binary gradient elution with a flow rate of 0.15 mL/min at 40°C. Eluent A was acetonitrile/water (1:9, v/v, J.T. Baker) and eluent B was isopropanol/acetonitrile (3:1, v/v, J.T. Baker); both eluents were supplemented with 10 mM ammonium acetate and 0.1% formic acid (Sigma-Aldrich). The following gradient conditions were used: 0 min, 40% B; 5 min, 70% B; 20 min, 90% B; 25 min, 100% B; 38 min, 100% B; 40 min, 40% B; 50 min, 40% B. The separated compounds were detected by using Agilent 6530 QTOF-MS. An electrospray source was operated in positive ionization mode. The ion source parameters were as follows: sheath gas temperature, 350°C; sheath gas flow, 12 L/min; nebulizer, 40 psi; dry gas temperature, 325°C; dry gas flow, 11 L/min; capillary voltage, 4000 V; nozzle voltage, 500 V; fragmentor, 100 V. Full scan data from 50–1,200 m/z was collected in centroid mode. The lock-mass reference ions (118.0863 and 922.0097 m/z) were delivered to the source continuously during data acquisition for mass calibration. Targeted MS/MS at collision energy of 20 eV was performed to identify all the lipids. The peak area from the extracted ion chromatogram (EIC) was measured by using MassHunter software (Agilent). The area was normalized to the internal standard for each species. The composition of the acyl chain in each lipid was measured by the EIC of [M-fatty acyl chain+H]^+^ fragment ion.

### Subcellular fractionation

For each group, liver tissues were pooled for subcellular fractionation. The liver tissues were homogenized in 1 mL buffer A containing 10 mM HEPES (pH 7.9), 1.5 mM MgCl2, 10 mM KCl, 0.5 mM DTT, and 0.05% NP40. The buffer was supplemented with protease inhibitors. The liver homogenates were centrifuged at 3,000 rpm for 10 min at 4°C; then the resulting supernatant was used as cytosolic sample. The pellet was re-suspended in 374 μL buffer B containing 5 mM HEPES (pH 7.9), 1.5 mM MgCl2, 0.2 mM EDTA, 0.5 mM DTT, 26% glycerol (v/v); and then 26 μl of 4.6 M NaCl. After homogenizing using syringe, it was on ice for 30 min. the suspended pellet was centrifuged at 24,000 g for 20 min at 4°C. The resulting supernatant was used as nuclei sample.

### Isolation of rat primary hepatocytes and cell culture

Male Sprague-Dawley rats were purchased from Japan SLC, Inc. (Hamamatsu, Japan). Primary hepatocytes were isolated from collagenase-perfused livers of male Slc:SD rats by a modification of a previously described method (21). The isolated cells were plated onto 6 cm dishes in 3 ml of DMEM supplemented with 5% FBS, 100 units/ml sodium penicillin, and 100 μg/ml streptomycin sulfate. After 4 h at 37°C incubator in 5% CO_2_, the attached cells were washed with PBS and then incubated in M199 supplemented with 100 nM dexamethasone and 1 nM insulin overnight as described in Chen et al. (22). And insulin was treated to a final concentration of 100 nM in rat primary hepatocytes.

### Statistical analysis

Data in the graphs were analyzed using the two-tailed Student's *t*-test for comparisons between two groups, or one-way ANOVA with the Tukey honest significant difference *post-hoc* test for multiple comparisons (SPSS 12.0K for Windows, SPSS, Chicago, IL) to determine statistical significance. A value of *P* < 0.05 was considered significant.

## Results

### Lobeglitazone ameliorates liver injury in response to physiological lipogenic stimulation

**Figure 1:**
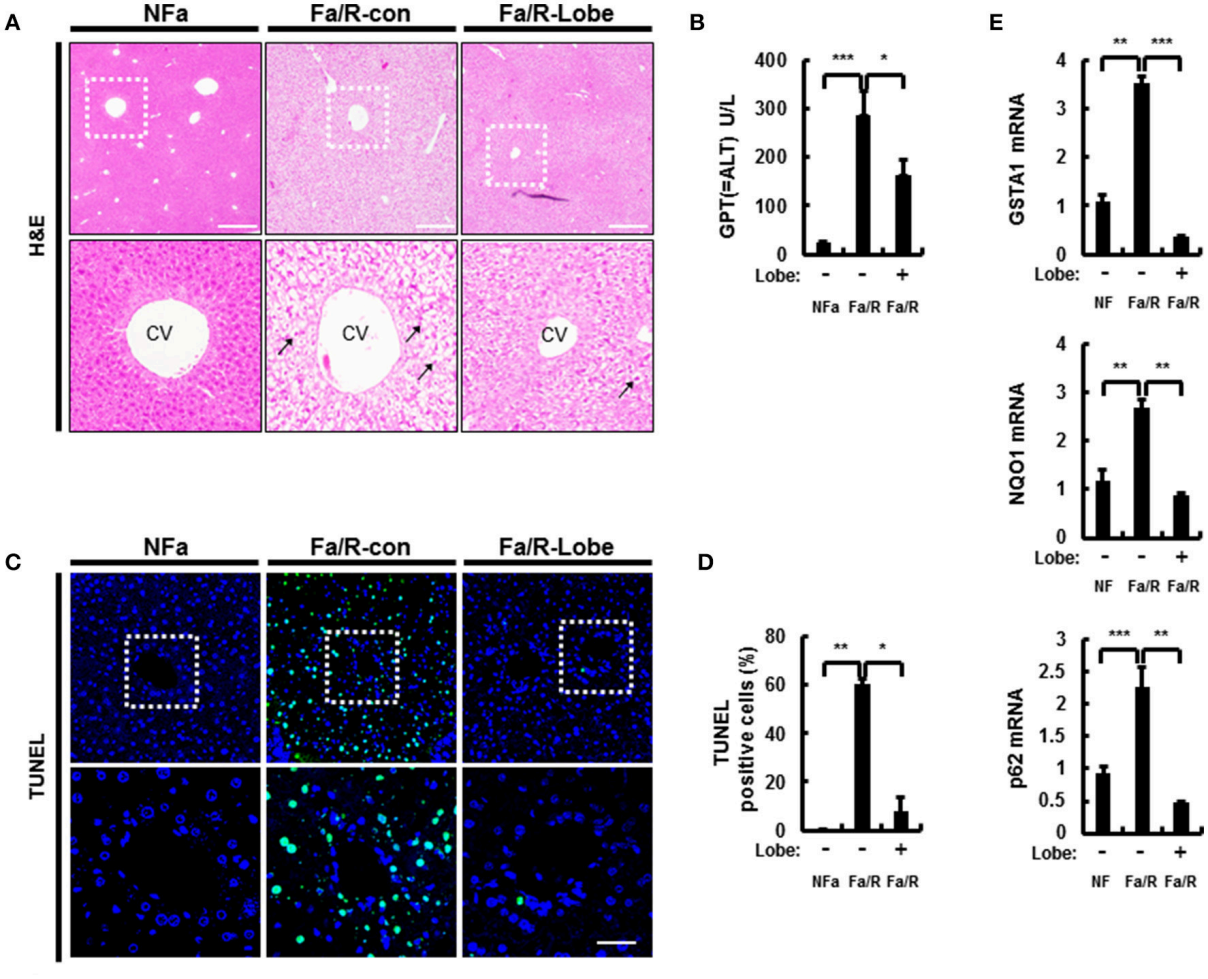
Lobeglitazone ameliorates acute lipogenic stimulus-mediated liver injury in mice. **(A–E)** Mice were maintained in a non-fasted state (NFa) (*n* = 8) or fasted overnight and then refed a high-carbohydrate, fat-free diet (Fa/R) with vehicle (*n* = 9) or lobeglitazone (*n* = 9). **(A)** The liver sections from mice were stained by using H&E. The extent of ballooning degeneration (arrow) is indicated on the bottom right set of images. CV, central vein. Scale bar = 200 μm. **(B)** The serum alanine aminotransferase (ALT) concentration was measured in each group of mice. **(C)** Images for TUNEL analysis of liver sections from each three groups of mice. Scale bar = 100 μm. **(D)** Quantitative graph of the TUNEL images of the liver sections. The data are the mean ± standard error for eight or nine mice of each group. *p < 0.05 and **p < 0.01. **(E)** Total RNA isolated from liver was subjected to qRT-PCR analysis of GSTA1 (top), NQO1 (middle), and p62 (bottom) mRNAs. The data are presented relative to the corresponding value for non-fasted mice and are the mean ± standard error for eight or nine mice in each group. *p < 0.05, **p < 0.01, and ***p < 0.001.

An increase of lipogenesis during the progression of NAFLD is one of the most important sources of fatty acids that contribute to the storage of TGs (23). Excess free fatty acids increase the susceptibility of the liver to oxidative stress. Thus, to examine whether lobeglitazone ameliorates hepatic injury, mice were fasted and then refed with a HCD, which are the physiological conditions that induce lipogenesis (7, 24). Subsequently, mice were injected with vehicle or lobeglitazone at 1 mg/kg by oral gavage before refed with HCD. We found that lobeglitazone alleviated refeeding-induced liver damage as measured by hematoxylin-eosin (H&E) staining (Figure 1A). In support of this finding, lobeglitazone decreased the increased ALT in physiological lipogenic stimulation (Figure 1B). To test whether lobeglitazone reduced liver injury-induced cell death, we stained a liver section of mice by using a terminal deoxynucleotidyl transferase dUTP nick end labeling (TUNEL) assay kit (Figures 1C,D). TUNEL-positive apoptotic cells were detected abundantly after refeeding, but this was significantly attenuated by lobeglitazone. The Nrf2 is master transcription factor for elimination of ROS. To further verify whether lobeglitazone attenuated the accumulation of reactive oxygen species (ROS) under physiological lipogenesis conditions, we examined the amount of nuclear Nrf2 by using nuclear fractionation (Supplementary Figures 1A,B). We found that lobeglitazone reduced translocation of Nrf2 into Nucleus, resulting in downregulation of its target genes (Figure 1E). We observed that lobeglitazone downregulated the activation of Nrf2 target genes induced in the adaptive response to oxidative stress. Collectively, these results indicated that lobeglitazone ameliorated oxidative stress-mediated liver injury through the inhibition of ROS generation.

### Lobeglitazone inhibits fatty acid ω-oxidation and ER stress

**Figure 2:**
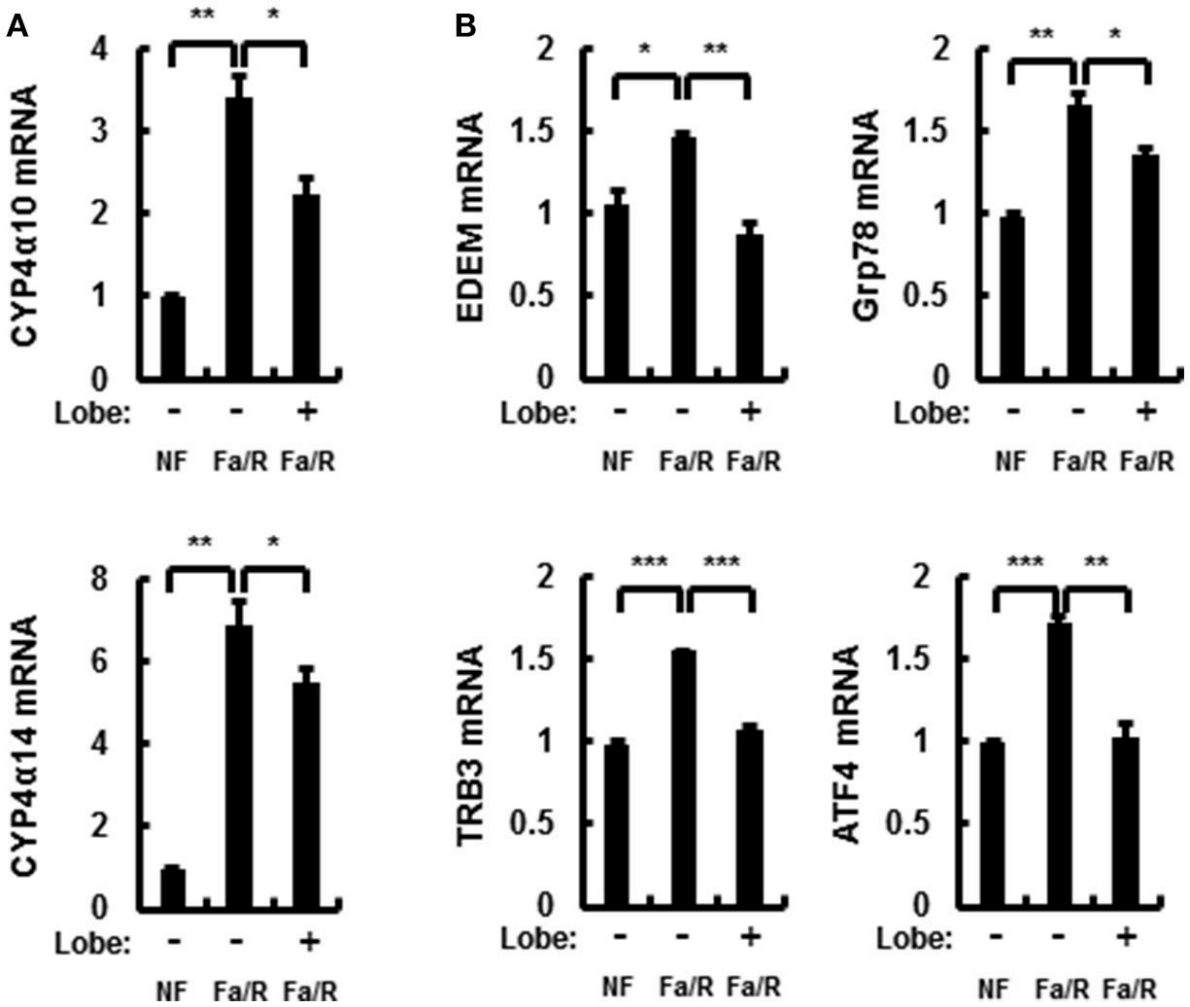
Lobeglitazone inhibits ER stress and fatty acid ω-oxidation upon acute lipogenic stimulus in mice. **(A,B)** Mice were maintained in a non-fasted state (NFa) (*n* = 8) or fasted overnight and then refed a high-carbohydrate, fat-free diet (Fa/R) with vehicle (*n* = 9) or lobeglitazone (*n* = 9). The isolated total RNA was subjected to qR, Grp78, TRB3, and ATF4 mRNAs **(B)**. The data are expressed relative to the corresponding value for non-fasted mice and are the mean ± standard error for eight or nine mice in each group. *p < 0.05, **p < 0.01, and ***p < 0.001.

Microsomal fatty acid ω-oxidation accelerates oxidative liver injury in NAFLD because it results in a dramatic generation of ROS (25). To explore whether lobeglitazone inhibited ω-oxidation-mediated ROS production, we evaluated the mRNA expression of cytochrome P450 enzymes 4A10 (Cyp4α10) and 4A14 (Cyp4α14) by using qRT-PCR analysis (Figure 2A). We found that lobeglitazone reduced the expression of ω-oxidation-related genes activated by refeeding. In addition, it has been reported that ROS was dramatically increased by ER stress in NAFLD and that refeeding with HCD in fasted mice induced hepatic ER stress (23, 24). Therefore, to investigate whether lobeglitazone inhibited ER stress, we evaluated the expression of ER stress-related genes, including ER degradation-enhancing α-mannosidase-like protein (EMDM), 78 kDa glucose-regulated protein (Grp78), tribbles pseudokinase 3 (TRB3), and activating transcription factor 4 (ATF4) and of CCAAT/enhancer-binding protein homologous protein (CHOP), ER stress-induced apoptosis marker, and found that lobeglitazone downregulated all the genes upregulated by refeeding (Figure 2B and Supplementary Figure 2). Taken together, these results suggested that lobeglitazone prevented liver injury by reducing fatty acid ω-oxidation and ER stress.

### Lobeglitazone inhibits hepatic lipid synthesis

**Figure 3:**
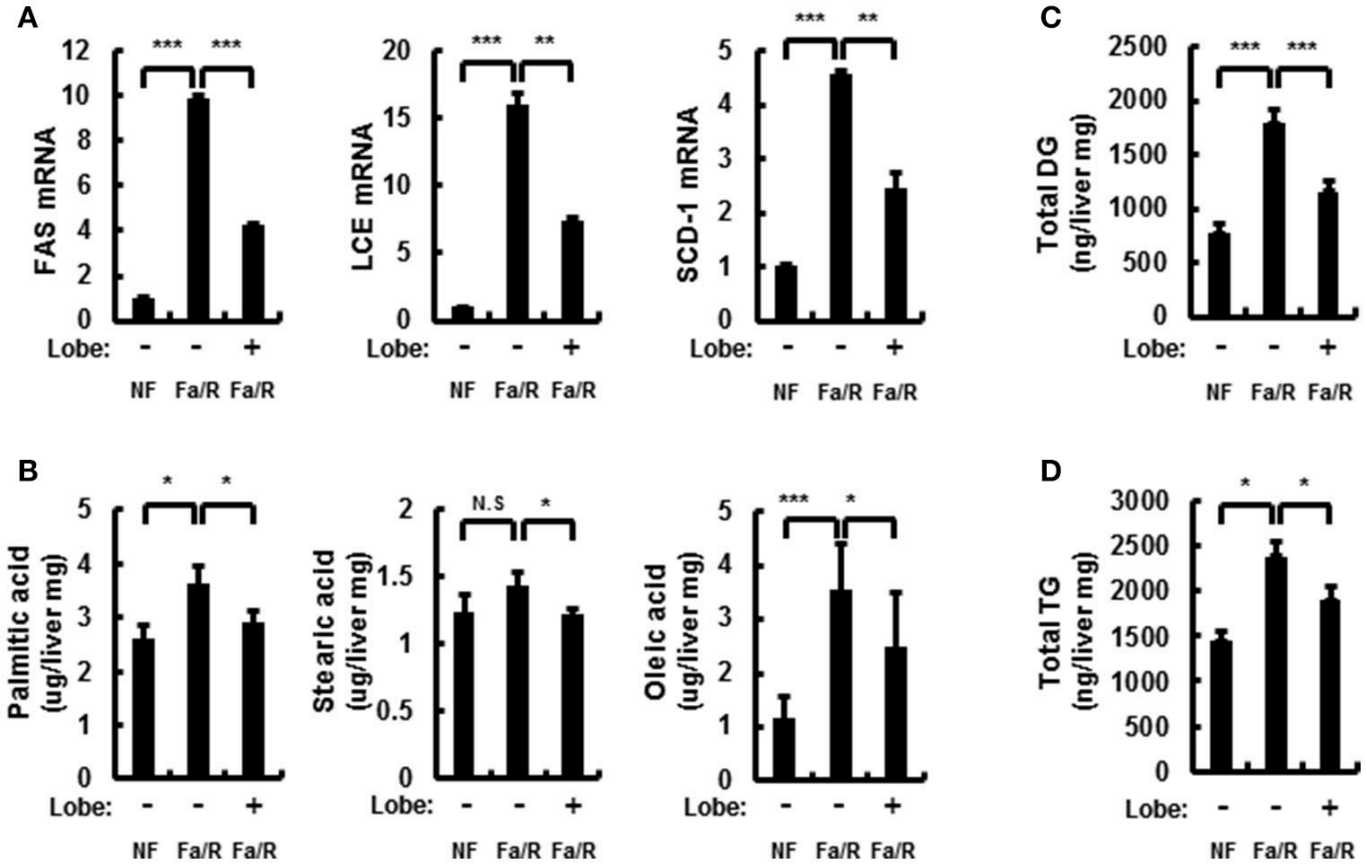
Lobeglitazone inhibits hepatic lipogenesis. **(A–D)** Mice were maintained in a non-fasted state (NFa) (*n* = 8) or fasted overnight and then refed a high-carbohydrate, fat-free diet (Fa/R) with vehicle (*n* = 9) or lobeglitazone (*n* = 9). **(A)** The total RNA isolated from liver was subjected to qRT-PCR analysis of FAS, SCD-1, and LCE mRNAs. The data are presented relative to the corresponding value for non-fasted mice. **(B)** The livers of eight or nine mice in each group were homogenized. The lipids from the homogenates were subjected to hydrolysis and acidic transmethylation, and then detected by GC-MS. Fatty acid content of palmitic acid, stearic acid, and oleic acid expressed per milligram of liver. **(C,D)** The livers of eight or nine mice in each group were homogenized. The lipids from the homogenates were subjected to hydrolysis and acidic transmethylation, and then detected by LC-MS. Lipid content of DG and TG expressed per milligram of liver. Data in **(A,D)** are the mean ± standard error for eight or nine mice in each group. *p < 0.05, **p < 0.01, and ***p < 0.001.

In the progression of NAFLD, the increase of lipid synthesis in the liver can promote ER stress and microsomal ω-oxidation. To investigate whether lobeglitazone regulated lipogenesis in the liver, we examined lipogenesis-related enzymes by using qRT-PCR and profiled the corresponding product by using GC-MS analysis (Figures 3A,B and Supplementary Figures 3A,B). We found that lobeglitazone reduced the mRNA expression of lipogenic genes induced by refeeding, as well as their products synthesized in the liver. Although ChREBP was not altered in fasting/refeeding, lobeglitazone reduced SREBP-1c and ChREBP, which are two transcription factors that regulate hepatic lipogenesis. We also examined the total amount of and concentrations of DGs and TGs, most of which are composed of two or three among these three fatty acids (Figures 3C,D and Supplementary Figures 3C,D). We showed that lobeglitazone significantly reduced the concentration of DGs and TGs under physiological lipogenic conditions. Increased malonyl-CoA inhibits β-oxidation through CPT-1 under acute lipogenic condition in hepatocyte; therefore, it accumulates lipid in the liver (26). To determine whether lobeglitazone affected fatty acid β-oxidation, we assessed the levels of carnitine palmitoyltransferase 1 (CPT-1) as measured by qRT-PCR analysis. We found that lobeglitazone increased the level of CPT-1, resulting in activation of mitochondrial β-oxidation in physiological lipogenic conditions. (Supplementary Figure 4). Collectively, these findings indicated that lobeglitazone inhibited lipid accumulation through a reduction in lipogenesis.

### Lobeglitazone inhibits the activation of the mTORC1 signaling pathway

**Figure 4:**
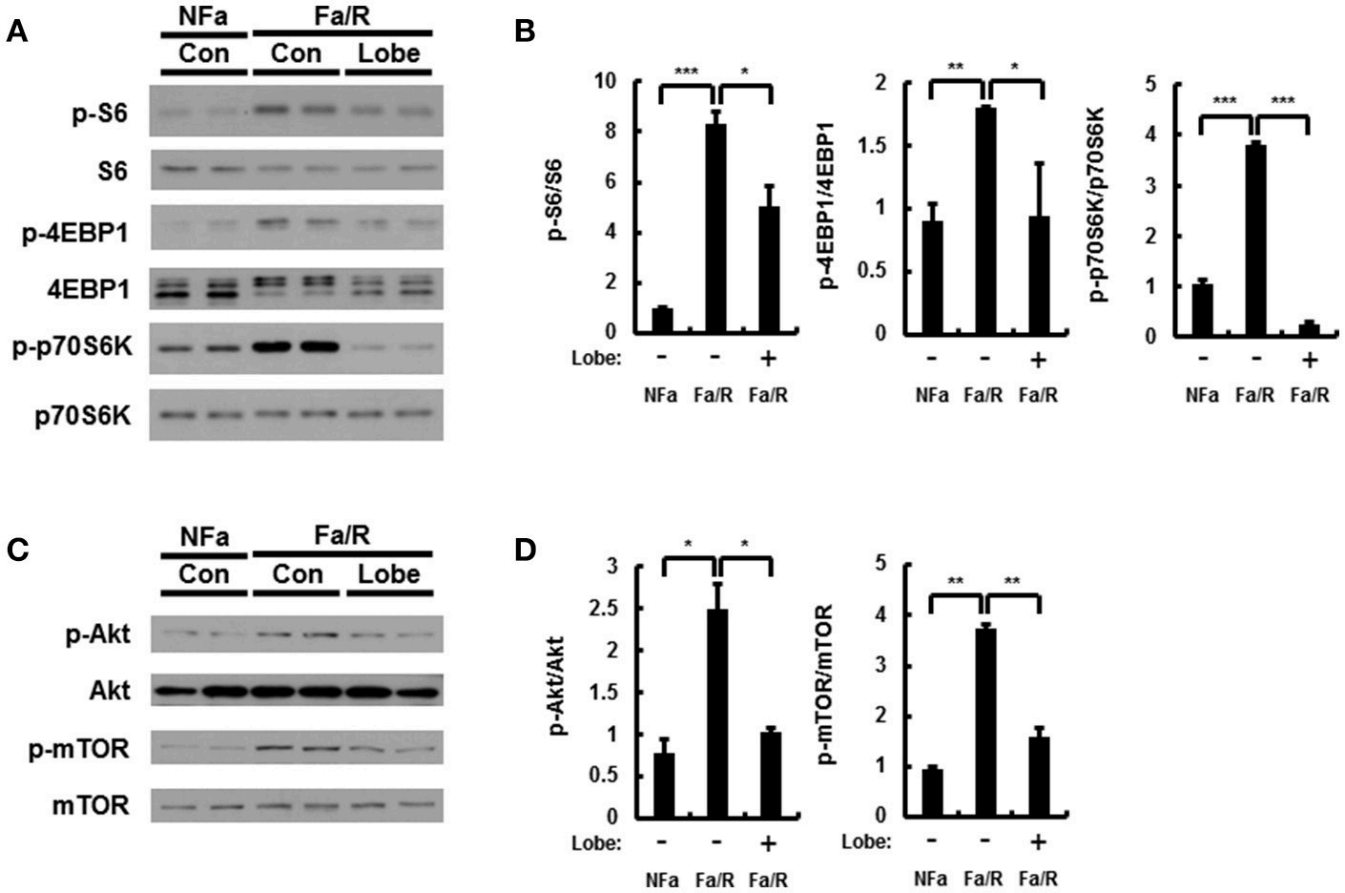
Lobeglitazone inhibits mTORC1 signaling induced by acute lipogenic stimulus in liver. **(A–D)** Mice were maintained in a non-fasted state (NFa) (*n* = 8) or fasted overnight and then refed a high-carbohydrate, fat-free diet (Fa/R) with vehicle (*n* = 9) or lobeglitazone (*n* = 9). **(A,C)** The livers from eight or nine mice of each group were pooled and homogenized; subsequently, the homogenates were subjected to immunoblotting analysis with antibodies to the indicated proteins. **(B,D)** The intensity of protein bands in **(A,C**, respectively) was determined by densitometry. The data in **(B,D)** are presented relative to the corresponding value for non-fasted mice and are the mean ± standard error for eight or nine mice in each group. *p < 0.05, **p < 0.01, and ***p < 0.001.

Hepatic lipid synthesis is induced by the hyperactivation of mTORC1 in NAFLD progression (7, 27, 28). Thus, to investigate whether lobeglitazone inhibited the activation of mTORC1, we analyzed the expression of mTORC1 signaling-related proteins by using immunoblotting (Figures 4A,B). It was identified that lobeglitazone downregulated the refeeding-induced phosphorylation of S6, p70S6K, and 4EBP1. Phosphorylated Akt can lead to activation of mTORC1 pathway by inducing phosphorylation of mTOR at serine 2448 (8). To further explore how lobeglitazone inhibited hyperactivation of mTORC1, we examined the levels of phosphorylation of Akt and mTOR as measured by immunoblot analysis (Figures 4C,D). We observed that under physiological lipogenic codintion, lobeglitazone reduced both phosphorylations of Akt and of mTOR in the liver. Activation of mTORC1 negatively regulates activity of PPARα, which induces fatty acid β-oxidation, and upregulates activity of PPARγ that control expression of genes involved in lipid synthesis (29). Consistent with these reports, we observed that lobeglitazone upregulated expression of PPARα while it reduced expression of PPARγ (Supplementary Figures 5A,B). Altogether, our results suggested that lobeglitazone protected the liver from lipogenesis-induced oxidative damage through the inhibition of Akt-mediated mTORC1 activation.

### Inhibiting the activation of the mTORC1 protects liver against oxidative injury from physiological lipogenic stimulation

**Figure 5:**
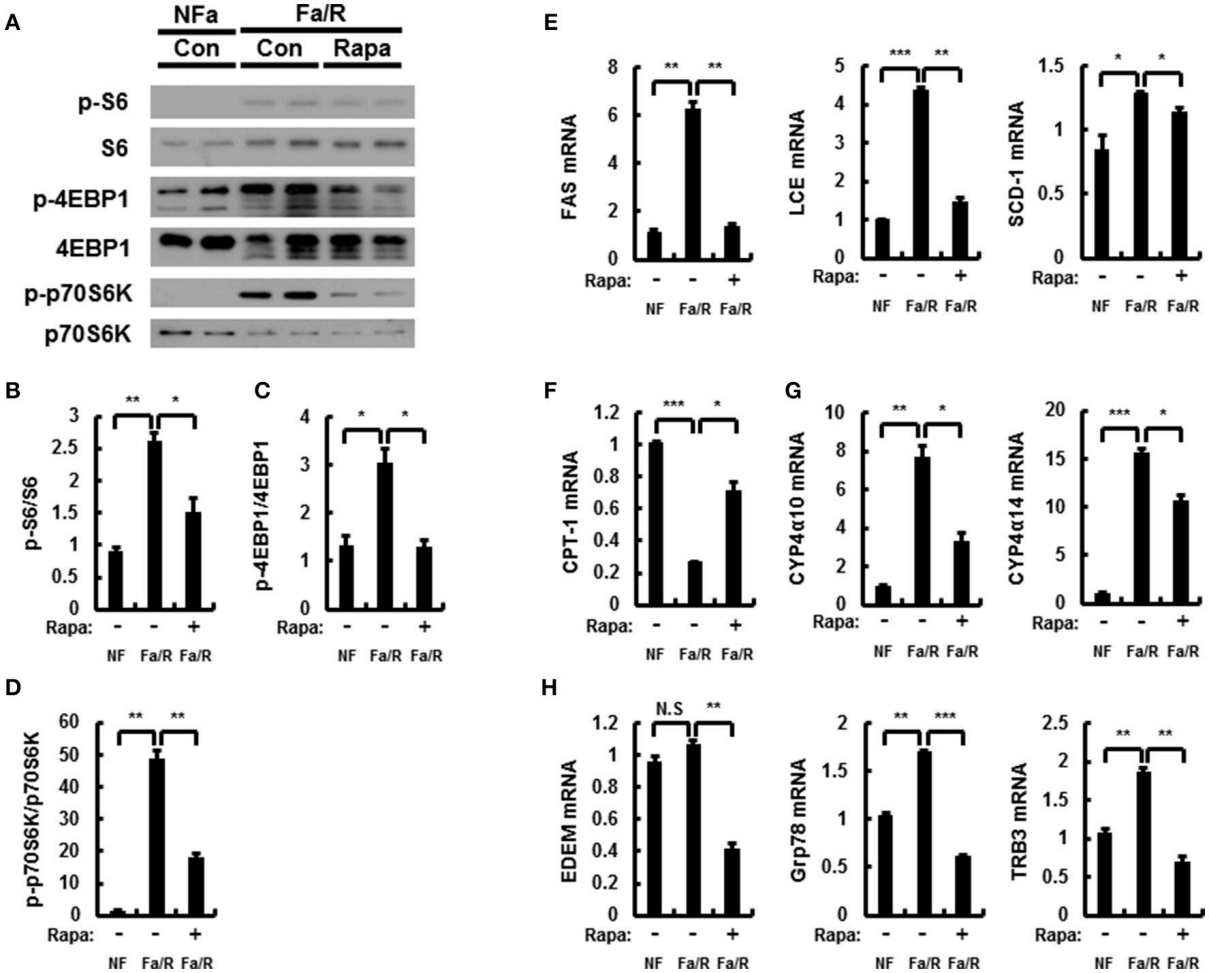
Rapamycin inhibits acute lipogenesis-induced oxidative stress in liver. **(A–H)** Mice were maintained in a non-fasted state (NFa) (*n* = 4) or fasted overnight and then refed a high-carbohydrate, fat-free diet (Fa/R) with vehicle (*n* = 4) or rapamycin (*n* = 4). **(A)** The livers from eight or nine mice of each group were pooled and homogenized; subsequently, the homogenates were subjected to immunoblotting analysis with antibodies to the indicated proteins. The intensity of protein bands to p-S6/S6 **(B)**, p-4EBP1/4EBP1 **(C)**, and p-p70S6K/p70S6K **(D)** in **(A)** was determined by densitometry. **(E–H)** The total RNA isolated from liver was subjected to qRT-PCR analysis. FAS, SCD-1, and LCE mRNAs **(E)**, CPT-1 mRNA **(F)**, Cyp4α10, and Cyp4α14 mRNAs **(G)**, and EDEM, Grp78, and TRB3 mRNAs **(H)**. The data are presented relative to the corresponding value for non-fasted mice. The data in **(B–H)** are presented relative to the corresponding value for non-fasted mice and are the mean ± standard error for eight or nine mice in each group. *p < 0.05, **p < 0.01, and ***p < 0.001.

Rapamycin inhibits mTORC1 signaling under acute lipogenic condition (8). To examine whether mTORC1 pathway inactivated by rapamycin reduces lipogenesis, ω-oxidation, and ER stress in this model, we subjected mice to fasting followed by refeeding with a HCD and subsequently injected with vehicle or rapamycin at 8 mg/kg by oral gavage before refeeding with HCD. And then, we analyzed the expression of mTORC1 signaling-related proteins by using immunoblotting (Figures 5A–D). It was identified that rapamycin inhibited activation of mTORC1 induced by refeeding with HCD. In addition, we evaluated expression of lipogenesis-, fatty acid oxidation-, and ER stress-related genes by using qRT-PCR analysis (Figures 5E–H). We showed that rapamycin inhibited expression of all genes except CPT-1 with inhibiting mTORC1 hyperactivation. Taken together, our data indicated that under acute lipogenic condition, inhibition of mTORC1 ameliorated not only increased lipogenesis, fatty acid ω-oxidation, and ER stress but also blocked fatty acid β-oxiation.

**Figure 6:**
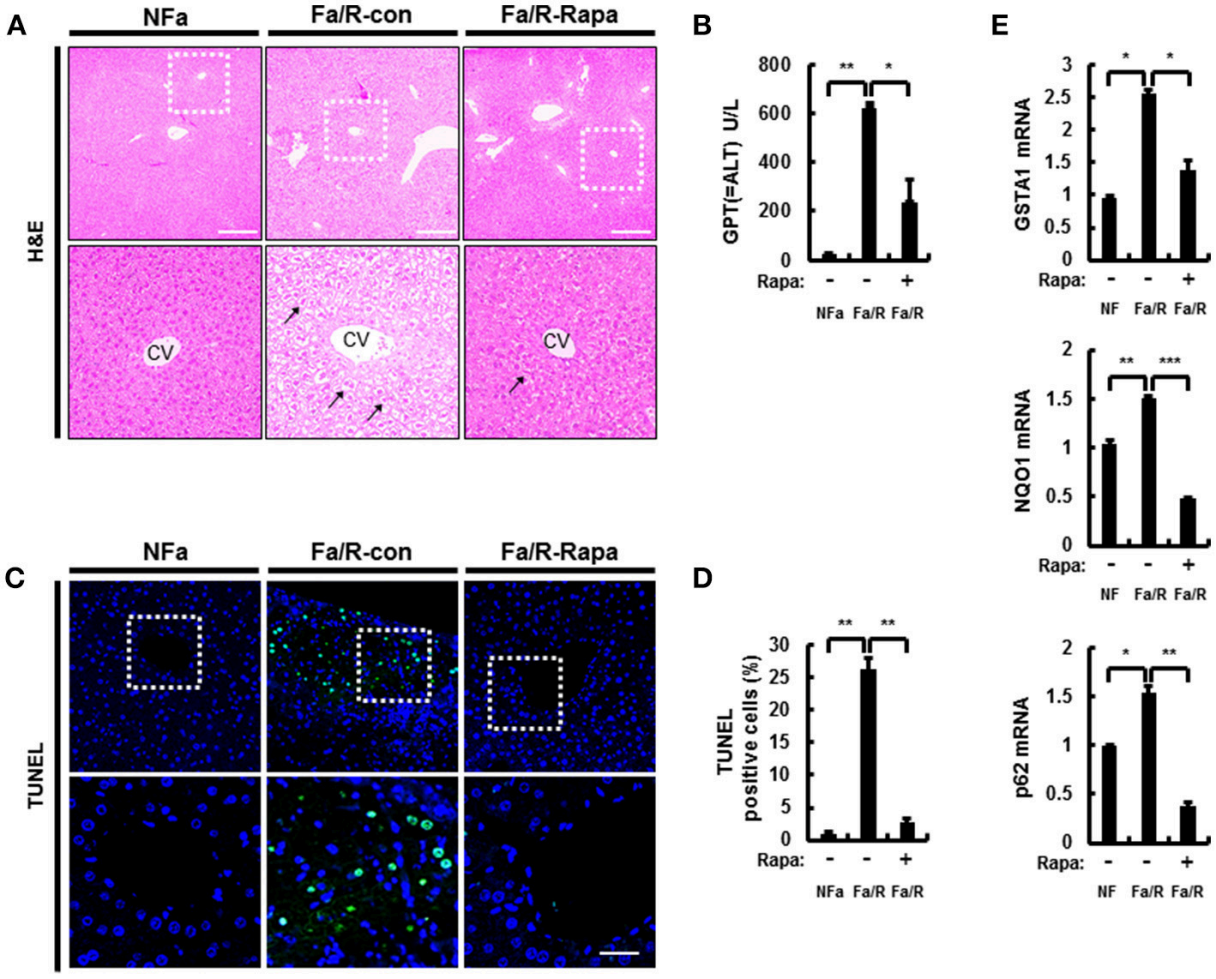
Inhibition of mTORC1 protects mice against acute lipogenic stimulus-mediated liver injury in mice. **(A–E)** Mice were maintained in a non-fasted state (NFa) (*n* = 4) or fasted overnight and then refed a high-carbohydrate, fat-free diet (Fa/R) with vehicle (*n* = 4) or rapamycin (*n* = 4). **(A)** The liver sections from mice were stained by using H&E. The extent of ballooning degeneration (arrow) is indicated on the bottom right set of images. CV, central vein. Scale bar = 200 μm. **(B)** The serum alanine aminotransferase (ALT) concentration was measured in each group of mice. **(C)** Images for TUNEL analysis of liver sections from each three groups of mice. Scale bar = 100 μm. **(D)** Quantitative graph of the TUNEL images of the liver sections. The data are the mean ± standard error for eight or nine mice of each group. *p < 0.05 and **p < 0.01. **(E)** Total RNA isolated from liver was subjected to qRT-PCR analysis of GSTA1 (top), NQO1 (middle), and p62 (bottom) mRNAs. The data are presented relative to the corresponding value for non-fasted mice and are the mean ± standard error for eight or nine mice in each group. *p < 0.05, **p < 0.01, and ***p < 0.001.

To further verify whether inactivation of mTORC1 by rapamycin improves acute lipogenesis-induced liver damage, we measured refeeding-induced liver damage by using hematoxylin-eosin (H&E) staining and serum ALT analysis (Figures 6A,B). We observed that rapamycin ameliorates liver injury in physiological lipogenic stimulation. Furthermore, we found that rapamycin reduced refeeding-induced cell death by using TUNEL assay kit (Figures 6C,D) In addition, we investigated whether rapamycin downregulated Nrf2 activation increased under this oxidative stress condition, we examined expression of Nrf2-dependent antioxidant genes, including GSTA1, NQO1, and p62 (Figure 6E). We found that rapamycin downregulated Nrf2 target genes. Collectively, our results suggested that inhibiting mTORC1 activation protected the liver from lipogenesis-induced oxidative damage.

### Lobeglitazone inhibits mTORC1 signaling in rat primary hepatocytes

**Figure 7:**
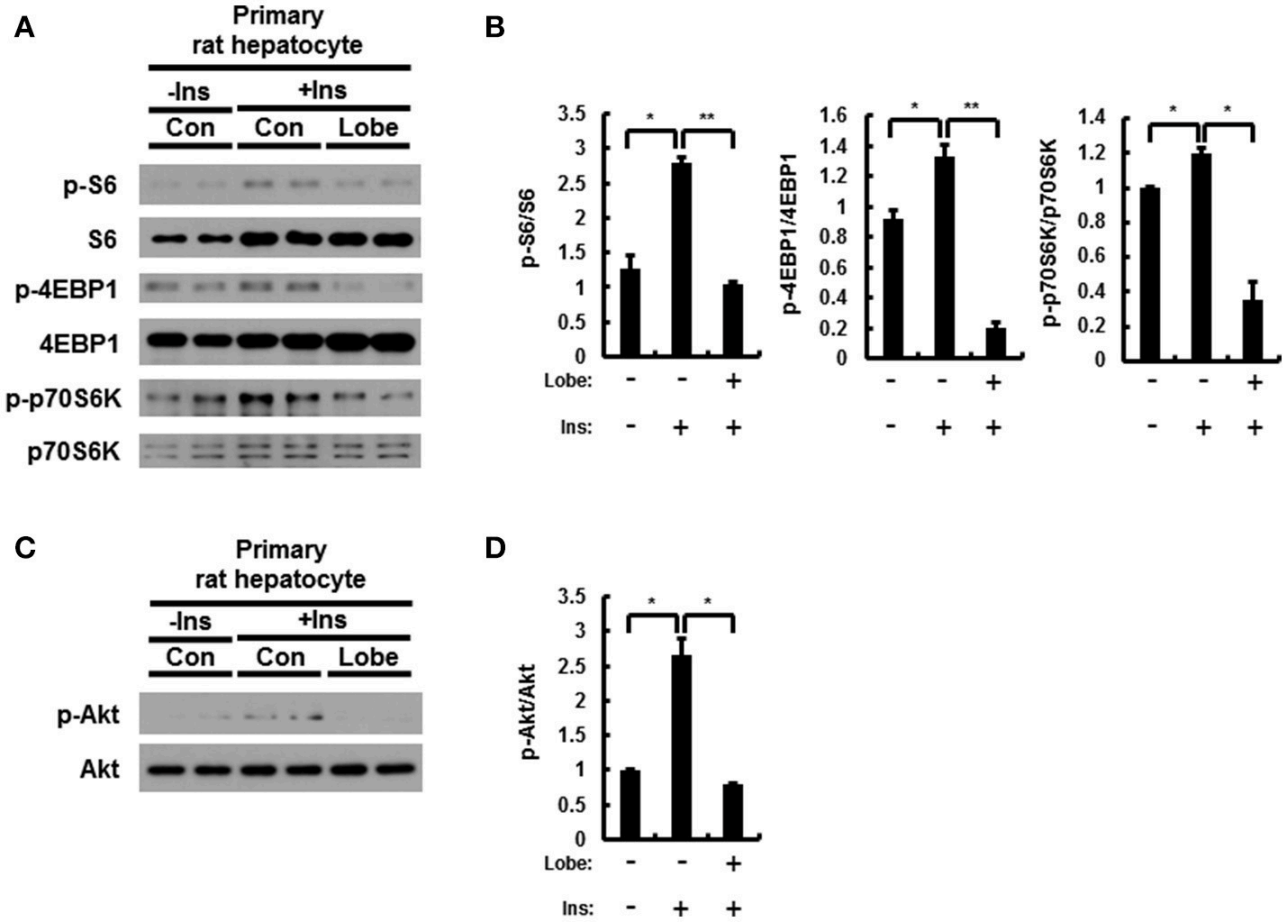
Lobeglitazone inhibits insulin mediated stimulation of mTORC1 signaling in rat primary hepatocyte. **(A–D)** The isolated cells were incubated in M199 supplemented with 100 nM dexamethasone and 1 nM insulin overnight, and then insulin was treated to a final concentration of 100 nM with or without lobeglitazone. **(A,C)** Primary hepatocytes isolated from rats were lysed and subjected to immunoblot anlaysis with antibodies to indicated proteins. **(B,D)** The intensity of protein bands in **(A,C**, respectively) and was determined by densitometry. Data in **(B,D)** are presented relative to the corresponding value for only vehicle-treated cells and are means ± Standard error for three independent experiments. *p < 0.05, **p < 0.01.

The mTORC1 signaling in rat primary hepatocytes treated with insulin is induced by increase of phosphorylation of Akt (8, 30). To investigate whether the effect of lobeglitazone on mTORC1 in the liver is primary effect, we isolated rat primary hepatocyte and treated insulin with or without lobeglitazone. As results in mice, mTORC1 signaling was enhanced in insulin-treated rat primary hepatocytes and alleviated in cells co-treated with lobeglitazone (Figures 7A,B). Furthermore, Lobeglitazone inhibited phosphorylation of Akt increased by insulin as in liver tissue of mice (Figures 7C,D). Taken together, these results suggested that lobeglitazone inhibits hepatic mTORC1 pathway via reducing phosphorylation of Akt in both *in vivo* and *in vitro*.

## Discussion

NAFLD is the most common chronic liver disease and is rapidly increasing worldwide (31). NAFLD has known as a risk factor for hepatic cirrhosis and hepatocellular carcinoma(32); however, there are no pharmacological therapies approved for treatment of NAFLD (10, 33).

**Figure 8:**
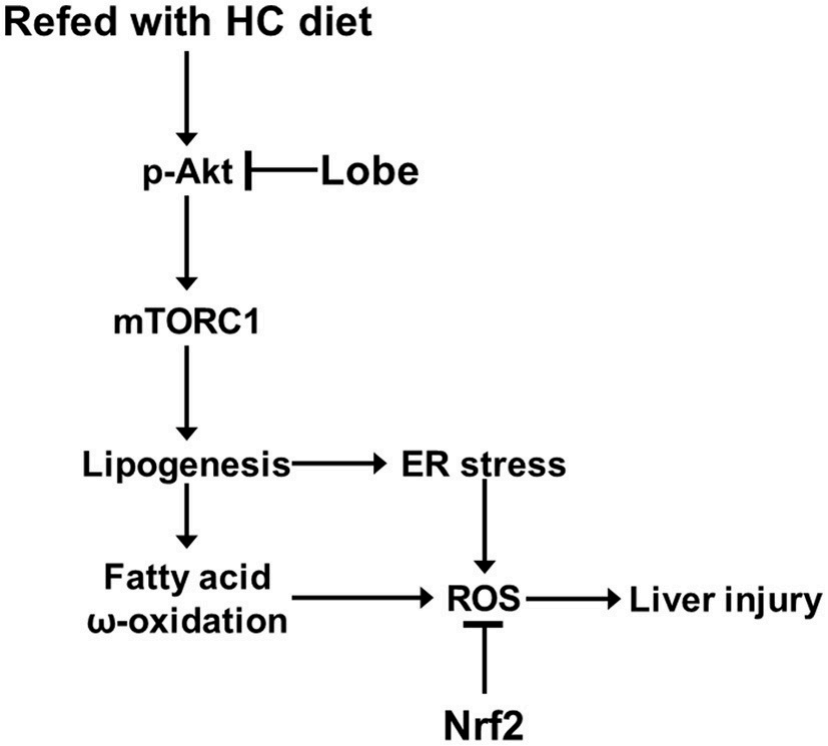
Model for mechanism of protective action of lobeglitazone against the liver injury. See text for details.

In recent studies, TZDs, insulin sensitizers, have been investigated as pharmacological therapy for treatment of NAFLD. Several studies reported that pioglitazone and rosiglitazone alleviated hepatic lipid accumulation and fibrosis in patients with NAFLD. Furthermore, pioglitazone promoted hepatic fatty acid oxidation in patients with NASH and reduced lipid synthesis in human muscle (34–37). It was proposed that TZDs ameliorated hepatic steatosis via the activation of the adiponectin-AMPK axis (38); but, pioglitazone and rosiglitazone have diverse side effects. Pioglitazone have the increased risks of bladder cancer and rosiglitazone is associated with myocardial infarction and cardiovascular mortality (39, 40); therefore, these are considered significant due to clinical uses. Recently, Lobeglitazone was developed as a more effective and safe TZD drug for treatment of type II diabetes (41). Lobeglitazone has similar efficacy in lipid metabolism to pioglitazone with even smaller dose in clinical studies (42, 43). In addition, lobeglitazone showed markedly reduction of adverse effects, such as cardiovascular disease and bladder cancer (44, 45). TZDs including lobeglitazone primarily act as PPARγ agonists in adipose tissue. As reported by several studies, pioglitazone, and rosiglitazone, increased uptake of fatty acids via activation of PPARγ in adipose tissue and consequently reduced free fatty acids in plasma, liver, or muscle. (46–48). Based on these reports, lobeglitazone, similar to other TZDs, may alleviate NAFLD development by increasing activity of PPARγ in adipose tissue. In addition, a recent study reported that lobeglitazone attenuated hepatic steatosis through the inhibition of PPARγ phosphorylation in chronic obese mice (17); however, underlying mechanism of the effects of lobeglitazone in NAFLD is still unclear. In our study, we investigated under physiological lipogenic condition, the effect of lobeglitazone and its molecular mechanism on the progression of NAFLD, with a focus on lipogenesis in the liver (Figure 8).

The mTORC1 signaling pathway is a central regulator of lipid metabolism (49, 50). The hyperactivation of mTORC1 increased the gene expression of SREBP-1c in the liver from patients with NAFLD (51). SREBP-1c is one of three SREBP isoforms and transcriptionally activates lipogenic genes (6). A recent study reported that the liver-specific knock-out of mTORC1 markedly impaired SREBP1 function and confers resistance to hepatic steatosis to mice (52). Thus, mTORC1 signaling is required for hepatic lipogenesis via the transcription of SREBP-1c (7, 8). Moreover, mTORC1 pathway upregulates peroxisome proliferator-activated receptor-γ (PPARγ) leading to lipogenesis and adipogenesis while negatively regulates peroxisome proliferator-activated receptor-α (PPARα) that induces fatty acid β-oxidation (29). Recent studies showed that activation of PPARγ induces hepatic steatosis, and that lipid accumulation is exhibited in PPARα-deficient mice (17, 53–57). Consequently, we found that lobeglitazone reduced expression of PPARγ and induced PPARα. Based on these findings, lobeglitazone is able to inhibit progression of NAFLD via suppression of PPARγ-induced adipogenic transformation of hepatocyte as well as acute lipogenesis-induced oxidative stress by inhibiting mTORC1 hyperactivation. The mTORC1 pathway can be regulated by Akt (58, 59). The phosphorylated Akt induces phosphorylation of mTOR at serine 2448 by inhibiting TSC2 or PRAS40, resulting in activation of mTORC1 pathway (8, 50, 60). Consistent with these reports, our results showed that lobeglitazone lead to inhibit Akt-dependent mTORC1 activation under acute lipogenic stimulation. Since the upstream events of the mTORC1 pathway are diverse, the reduction of phosphorylation of Akt by lobeglitazone may be one of those events. More detailed mechanisms should be studied in the future.

An increase in hepatic lipogenesis by hyperactivation of mTORC1 promotes lipid deposition and oxidative stress-induced liver injury (1, 61). Free fatty acids increased by hepatic lipogenesis were converted to TG and incapable of mitochondrial β oxidation through the inhibition of CPT-1, resulting in fatty acid overload in hepatocytes (5, 62). Consequently, saturated and unsaturated fatty acids may go through microsomal ω-oxidation by CYP4A enzymes. Although ω-oxidation in microsome is a minor pathway of fatty acid metabolism, it significantly can be increased in liver overloaded with fatty acids (25). Fatty acid ω-oxidation generates ROS, which is the major microsomal sources of oxidative stress in NAFLD (63). Here, we found that lobeglitazone inhibited the expression of ω-oxidation-related genes.

Furthermore, fatty acid overload in the liver modifies the lipid composition of ER membrane, which triggers ER stress (1, 23, 64). The increased ER stress in NAFLD progression not only acts as another inducer of oxidative stress, but also plays a causative role in the process of lipogenesis (27, 65). In our findings, lobeglitazone reduced ER stress via inhibiting lipid synthesis. Nrf2 is known as the master transcription factor for the antioxidant response, and can attenuate oxidative stress-induced liver injury by eliminating accumulated ROS (66, 67). Our results showed that lobeglitazone prevented the generation of ROS through inhibition of ω-oxidation and ER stress, resulting in reduction of Nrf2 target genes under acute lipogenic condition. Consequently, lobeglitazone protects the liver from oxidative stress-mediated injury. Collectively, these results support that lobeglitazone exerts hepatoprotective effects by inhibiting hepatic lipogenesis as well as increasing insulin sensitivity; thus, we have suggested that lobeglitazone may be clinically used as a therapeutic drug for NAFLD.

## Author contributions

YL carried out the experiment and wrote the manuscript with support from JP. DL contributed to animal experiment. D-KL and SK performed lipid profiling. B-WL helped the preparation of the chemicals SB supervised the project. All authors helped shape the analysis, research, and manuscript.

### Conflict of interest statement

The authors declare that the research was conducted in the absence of any commercial or financial relationships that could be construed as a potential conflict of interest.

## Abbreviations

NAFLD: non-alcoholic fatty liver disease
T2D: type II diabetes
mTORC1: mechanistic target of rapamycin complex 1
TZD: thiazolidinedione
ER: endoplasmic reticulum
NASH: non-alcoholic steatohepatitis
SREBP-1c: sterol regulatory element-binding protein-1c
ChREBP: carbohydrate response element binding protein
HCD, high-carbohydrate diet: fat-free diet
ALT: alanine aminotransferase
DG: diglyceride
TG: Triglyceride
ROS: reactive oxygen species
GSTA1: glutathione S-transferase A1
NQO1: NAD(P)H quinone dehydrogenase 1
Cyp4α10: cytochrome P450 enzymes 4A10
Cyp4α14: cytochrome P450 enzymes 4A14
EMDM: ER degradation-enhancing α-mannosidase-like protein
Grp78: 78 kDa glucose-regulated protein
TRB3: tribbles pseudokinase 3
ATF4: activating transcription factor 4
FAS: fatty acid synthase
ACC: acyl coenzyme A carboxylase
SCD-1: stearoyl CoA desaturase 1.
