# Supplementary material for: The Antidiabetic Drug Lobeglitazone Protects Mice From Lipogenesis-Induced Liver Injury via Mechanistic Target of Rapamycin Complex 1 Inhibition
Source: Front Endocrinol (Lausanne). 2018 Sep 21;9:539. doi: 10.3389/fendo.2018.00539 (PMC6161559; doi:10.3389/fendo.2018.00539)
Supplement: Supplementary file 1 [file Data_Sheet_1.docx]

**Supporting Information**

**The Antidiabetic Drug Lobeglitazone Protects Mice from Lipogenesis-Induced Liver Injury via Mechanistic Target of Rapamycin Complex 1 Inhibition**

Yu Seol Lee^1,2^, Jeong Su Park^2^, Da Hyun Lee^1,2^, Dong-Kyu Lee^3^, Sung Won Kwon^3,4^, Byung-Wan Lee^5^ and Soo Han Bae^2*^

^1^Brain Korea 21 PLUS Project for Medical Science, Yonsei University;

^2^Severance Biomedical Science Institute, Yonsei Biomedical Research Institute, Yonsei University College of Medicine, 50 Yonsei-ro, Seodaemun-gu, Seoul 03722, Republic of Korea;

^3^ Research Institute of Pharmaceutical Sciences, Seoul National University, Seoul, 08826, Republic of Korea

^4^ College of Pharmacy, Seoul National University, Seoul, 08826, Republic of Korea

^5^Department of Internal Medicine, Yonsei University College of Medicine, Seoul, Korea

Corresponding authors:

Soo Han Bae, Ph.D.

soohanbae@yuhs.ac

**Supplementary Figure Legend**

**Supplementary Fig. 1. Lobeglitazone downregulates Nrf2 activation.**

(A and B) Mice were maintained in a nonfasted state (NFa) (n=8) or fasted overnight and then refed a high-carbohydrate, fat-free diet (Fa/R) with vehicle (n=9) or Lobeglitazone (n=9). (A) The livers from eight or nine mice of each group were pooled and homogenized; and then the homogenates were subjected to nuclear fractionation and immunoblot analysis with antibodies to the indicated proteins. (B) The intensity of protein bands in (A) was determined by densitometry. Data were expressed relative to the corresponding value for nonfasted mice and are means ± Standard error for eight or nine mice of each group. *p < 0.05.


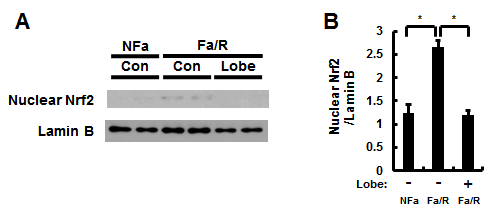


**Supplementary Fig. 1.**

**Supplementary Fig. 2. Lobeglitazone reduces ER stress.**

Mice were maintained in a nonfasted state (NFa) (n=8) or fasted overnight and then refed a high-carbohydrate, fat-free diet (Fa/R) with vehicle (n=9) or lobeglitazone (n=9). Total RNA isolated from the liver was subjected to qRT-PCR analysis for CHOP mRNA. The data are presented relative to the corresponding value for nonfasted mice and are the mean ± standard error for eight or nine mice in each group. ^*^p < 0.05 and ^**^p < 0.01.


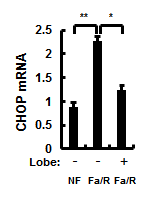


**Supplementary Fig. 2.**

**Supplementary Fig. 3. Lobeglitazone suppresses lipogenesis.**

(A-D) Mice were maintained in a nonfasted state (NFa) (n=8) or fasted overnight and then refed a high-carbohydrate, fat-free diet (Fa/R) with vehicle (n=9) or lobeglitazone (n=9). (A and B) Total RNA isolated from the liver was subjected to qRT-PCR analysis for ACC1 and GPAT mRNA (A) and SREBP-1c and ChREBP mRNA (B).The livers of eight or nine mice in each group were homogenized. Major diglycerides (DGs) (C) and triglycerides (TGs) (D) of the homogenates were detected by LC-MS. The data in (A-D) are presented relative to the corresponding value for nonfasted mice and are the mean ± standard error for eight or nine mice in each group. ^*^p < 0.05, ^**^p < 0.01, and ^***^p < 0.001.


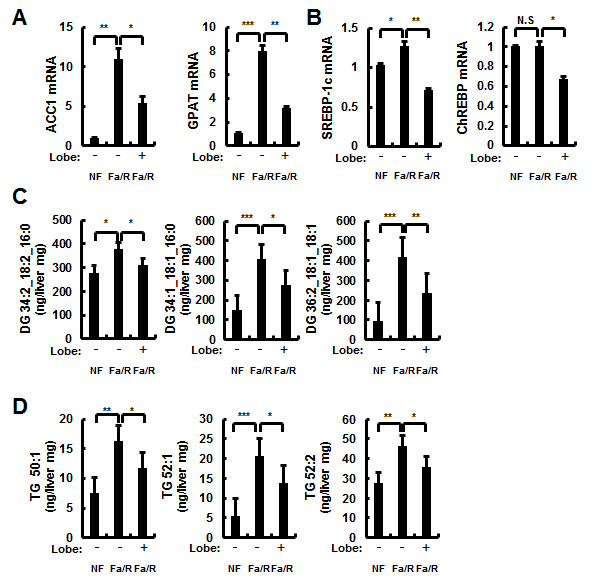


**Supplementary Fig. 3.**

**Supplementary Fig. 4. Lobeglitazone increases fatty acid β-oxidation.**

Mice were maintained in a nonfasted state (NFa) (n=8) or fasted overnight and then refed a high-carbohydrate, fat-free diet (Fa/R) with vehicle (n=9) or lobeglitazone (n=9). Total RNA isolated from the liver was subjected to qRT-PCR analysis for CPT-1 mRNA. The data are presented relative to the corresponding value for nonfasted mice and are the mean ± standard error for eight or nine mice in each group. ^**^p < 0.01 and ^***^p < 0.001.


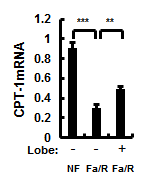


**Supplementary Fig. 4.**

**Supplementary Fig. 5. Lobeglitazone regulates activity of PPARα and PPARγ.**

(A and B) Mice were maintained in a nonfasted state (NFa) (n=8) or fasted overnight and then refed a high-carbohydrate, fat-free diet (Fa/R) with vehicle (n=9) or lobeglitazone (n=9). Total RNA isolated from the liver was subjected to qRT-PCR analysis for PPARα (A) and PPARγ (B) mRNA. The data are presented relative to the corresponding value for nonfasted mice and are the mean ± standard error for eight or nine mice in each group. ^*^p < 0.05.


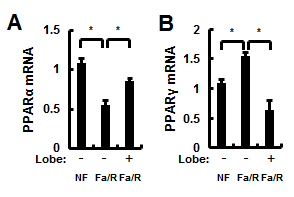


**Supplementary Fig. 5.**
